# Possibilities of reducing the number of welds on rail vehicle doors

**DOI:** 10.1038/s41598-022-20837-w

**Published:** 2022-10-07

**Authors:** Marian Sigmund, Jan Spichal

**Affiliations:** Faculty of Mechanical Engineering, Institute of Welding and Surface Treatment, Brno University of Technology, Brno, Czech Republic

**Keywords:** Mechanical engineering, Metals and alloys

## Abstract

The paper developed methods that can be employed to reduce the number of welds on a specific rail-vehicle frame door welded from the EN AW 6060 aluminum alloy profiles thermally processed into the T66 state. The profiles were welded by the GTAW method using an S Al 5087 (AlMg4,5MnZr) wire as the filler material. Tensile tests were performed on the supplied samples after welding to check the mechanical properties required. The resulting tensile test data were subsequently used as boundary values for a new design of the door frame having fewer welds. A FEM simulation was carried out using the Virtual Performance Solution software with PAM—Crash extension. The study's biggest achievement was reducing two welds on a real frame door without changing door frame stability. In view of saving welding and producing time and finance by reducing the number of loaded welds. In conclusion, this designed variant is evaluated and tested.

## Introduction

During manufacturing of door systems for trains or vehicles is suitable selecting proper and suitable primer construction. Due to its excessive segmentation and specific customer needs, it is impossible or causing complex problems and increased costs to produce aluminium frame doors without welding. The input materials include metallurgical intermediary products that must meet requirements of the design (strength, toughness), technology (formability, weld ability), as well as economy. Increasing passenger volumes and a progressing use of public transport require highly reliable entrance systems. Rolling stock manufacturers, systems suppliers and train operators are facing the technical challenge to meet the continuously rising requirements for safety, passenger comfort and barrier-free use for persons with reduced mobility^1^. Welding technology is one of the main joining methods and widely used in industries to assemble various products such as ships, vehicles, trains. For instance, the assembly process in train building essentially involves the joining of large blocks. Typically, these blocks are all-welded, thin-plate structures for example train door frame system. Welding induced distortion not only degrades the performance but also increases the cost of a fabricated structure. Therefore, it is very important if it is possible reduce welding. Or for aluminum structures is possible change welding to brazing or bonding. Generally, there are two major causes of geometrical error in the welded structure^2^. The first is local shrinkage due to rapid heating and cooling in the weld zone. Basically, local shrinkage can be divided into three categories: longitudinal shrinkage, transverse shrinkage and angular distortion. These three kinds of local deformations can be taken as inherent deformations^3^. They are strongly influenced by heat input, shape of penetration, plate thickness and joint type. The second is root gap and misalignment produced in the joint before or during welding. Contributing factors to gap and misalignment are initial geometrical error, welding sequence, positioning, restraint and tack weld. In order to precisely and reliably predict the welding deformation during assembly process, all these factors must be taken into account in addition to the local shrinkage^4^. During welding, the heat input must be kept at a low range, to ensure less internal stress and deformation being created. It also minimizes or eliminates the necessity of a subsequent straightening and heat treatment of the weldment. There are several possibilities of reducing the heat input such as changing the welding method or automatization of used welding method to higher welding speed, adjusting the technological process, and reducing or completely cancelling welds in some FEM defined places^5^. Predictive fracture mechanics within numerical codes has become a necessity for the virtual design of an automotive and train vehicles but at the same time it is still very difficult to achieve. An engineering structures, especially aluminum structures of train vehicles, usually contain flaws or micro cracks. Consequently, engineering design often requires evaluation of the maximum flaw size and operating stress level for safe operation. Large flaws or high stresses can lead to crack growth and ultimately to unstable propagation and structural failure. Knowledge of the “fracture toughness” (in general terms) of a material, i.e. the measure of its resistance to crack growth, is required to design against unstable crack propagation^6,7^.

## Welded part

**Figure 1:**
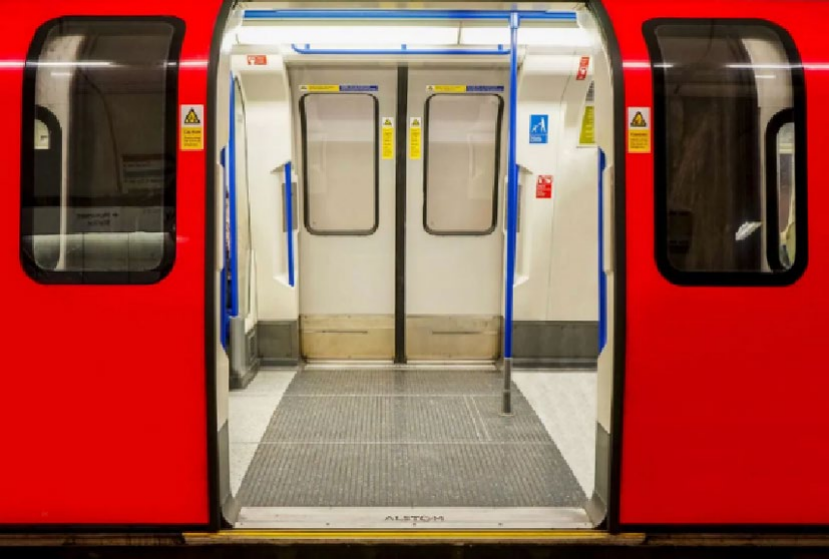
Door system.

**Figure 2:**
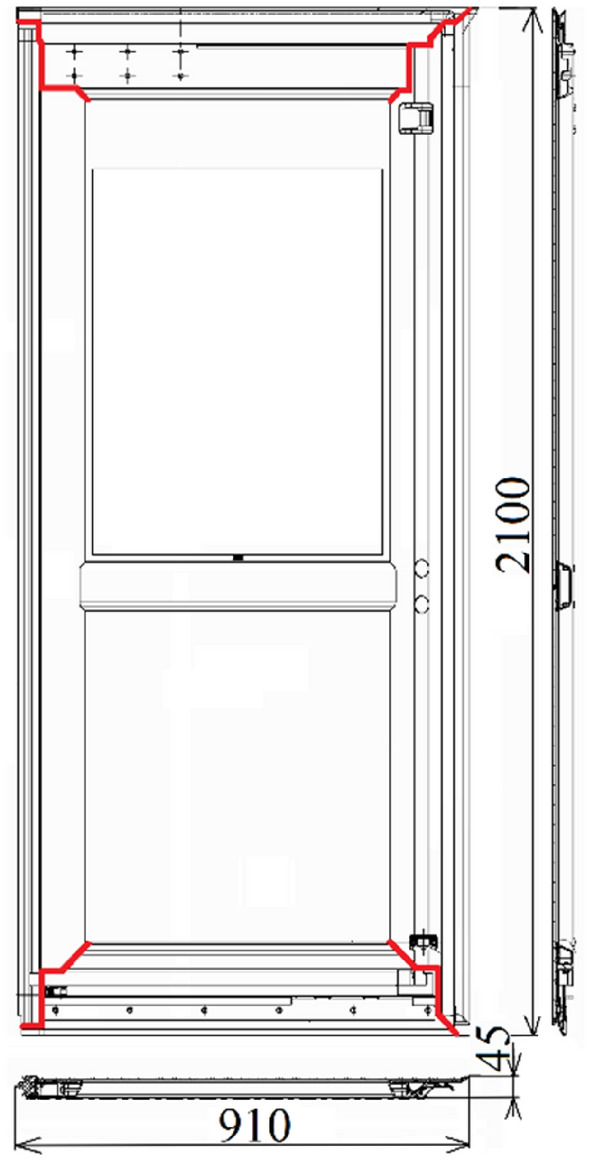
Door frame.

A door system consists of three elements: door, opening mechanism, and control section. The control section is the brain of the door system that controls and monitors the door to secure correct operation while providing maximum safety. This is ensured by numerous transmitters and sensors, which continually monitor the door area. It is possible to select a pick-up transmitter opening and shutting the door, safety relay in the motor driver, signal notation, acoustic or visual, photographic cells with door-latching feedback, sensitive edges with grind or traction detection. Very useful is also so-called artificial wear memory, which, based on the door wear, after a time, itself reacts to a change in the power needed or the door-opening speed. These aspects are compensated for to prevent unnecessary error messages and to reduce the necessary servicing. Figure 1 shows the complete door system. Figure 2 shows a specific door frame^1^.

## Basic material and welding technology

The design of the door frames consists of aluminous profiles welded together. To reduce the number of welds necessary and the heat input, thin walled profiles are used with excessively complex shapes. Much emphasis is put on the formability and weld ability of the basic material. Next, corrosion resistance is required, as well as low specific weight, low purchase price, and high strength. These characteristics are guaranteed by the EN AW 6060 aluminium alloy. Magnesium and silicon are the primary alloying elements, but it also contains further alloy additions improving its characteristics.

### The basic material used

The aluminous profiles supplied from the EN AW 6060 material are thermally treated into a T66 state. By this type of heat treatment, it is possible to achieve the best mechanical properties of this material. According to the EN 515 standard, this state is defined as a state after solution annealing with the resulting artificial aging—the mechanical properties are better than those for the T6 state, which is achieved by a specifically controlled treatment. The solvent temperature of the inter-metallic phase Mg_2_Si ranges between 500 and 540 °C. It is magnesium and silicon that influence this temperature most of all^8^. Because of the small content of the chief alloying elements, the alloy is “self-hardening", that is, it is possible to obtain shot solid solution during air cooling. This is a big advantage, because, during the cooling, aluminous profiles are subject to less deformation not necessitating subsequent straightening. The resulting aging proceeds at a temperature of 160–180 °C for a period of 8–12 h, in which the alloying elements are diffused and the Mg2Si phase is nucleated^9^. The below Tables 1 and 2 show the chemical composition of the EN AW 6060 T66 aluminous alloys and the tensile properties of the basic material.

**Table 1:** Chemical composition of the EN AW 6060 T66 aluminous alloy.

Si (%)	Fe (%)	Cu (%)	Mn (%)	Mg (%)	Cr (%)	Ni (%)
0.42	0.19	< 0.01	0.05	0.42	< 0.01	0

Zn (%)	Ti (%)	Al (%)				
< 0.01	0.01	Rest

**Table 2:** Tensile properties of the EN AW 6060 T66 aluminous alloy.

R_p0.2_ (MPa)	R_m_ (MPa)	A (%)
200.6	225.5	11.5

### The filler material used

Solid wire classified as S Al 5087 is used as a filler material for the door frame project. The zirconium alloying element improves the fracture strength while the weld metal solidifies preventing hot cracking. The chemical composition of the filler material and the tensile properties of the test report 3.1 are displayed in Tables 3 and 4.

**Table 3:** Chemical composition of the S Al 5087 filler material.

Si (%)	Fe (%)	Cu (%)	Mn (%)	Mg (%)	Cr (%)	Ni (%)
0.02	0.09	< 0.01	0.8	4.6	0.07	0

Zn (%)	Ti (%)	Al (%)	Zr (%)	Be (%)		
< 0.01	0.11	94.1	0.12	0.0003

**Table 4:** Tensile properties of the S Al 5087 filler material.

R_p0.2_ (MPa)	R_m_ (MPa)	A (%)
210	280	18

### The welding technology used

The test piece (3 mm thick plate) from the EN AW 6060 T66 aluminous alloy was welded with butt weld using the GTAW (141 according to ISO 4063) welding method. The welding was done by the appropriate welding procedure specifications. As the filler material, wire with a diameter of 2.4 mm was used classified as S Al 5087 (AlMg4,5MnZr). The welding parameters are shown in Table 5.

**Table 5:** Welding parameters.

Base current Iz (A)	Impulse current Ip (A)	Welding voltage U (V)	Welding speed v (cm/min)	Heat input Q (kJ/cm)
180	160	14.5	20	4.49

**Figure 3:**
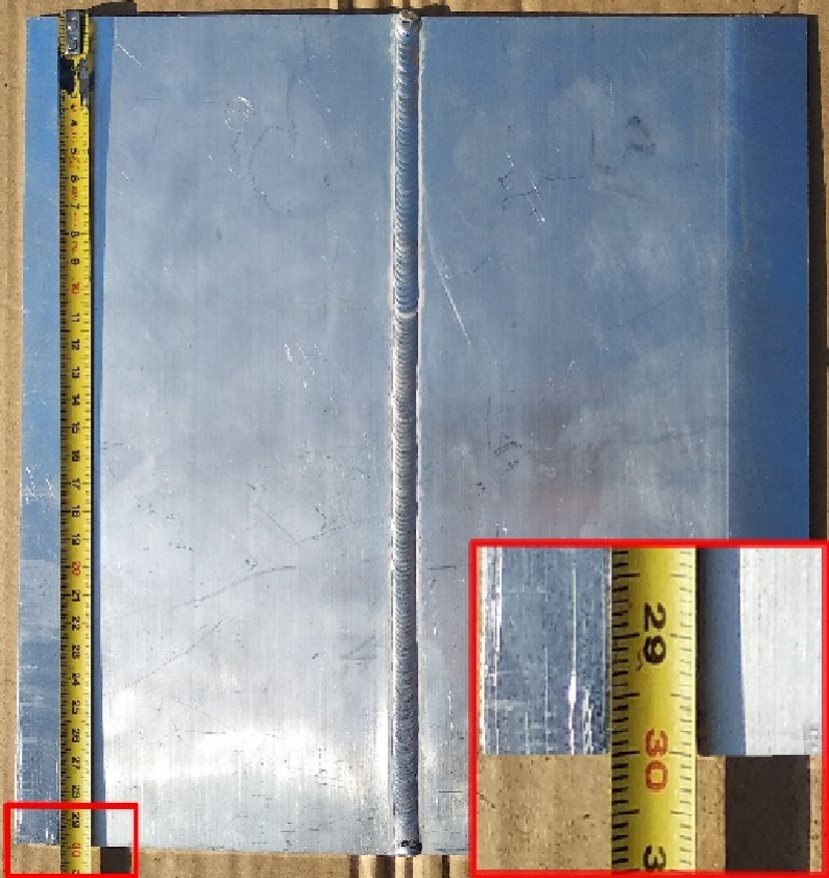
Welded aluminous alloy plate.

As a shielding gas, I3—Ar/He 30 was used with a gas-flow rate of l = 13–15 L per minute. The time of the first blow was set to 1 s and the time after the blow was set to 3 s. The test piece was welded without oscillation by a WCe tungsten electrode with a diameter of diameter 2.4 mm. The welded aluminous alloy plate is shown in Fig. 3.

### The test specimen made for tensile test

**Figure 4:**
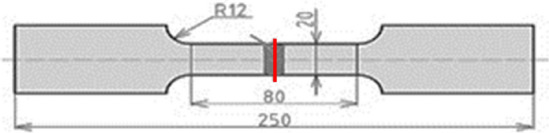
Test specimen for tensile test.

The test specimens for tensile tests were made from the welded aluminium alloy plates. A tensile test was performed according to the EN ISO 6892-1 standard. The final outline design for the tensile test is shown in Fig. 4. In producing the specimen, it is important to ensure that the aluminium alloy material should not be thermally influenced at all or only to a small extent. An increased temperature would set off a precipitation hardening process changing the mechanical properties and, thus, the required yield strength. The yield strength measured would differ from the properties of the real door frame. Even the best FEM simulation would lead to wrong calculations and wrong results. In the end, the aluminous alloy plate was cut into stripes each of them being subsequently milled.

### The tensile test evaluation

**Figure 5:**
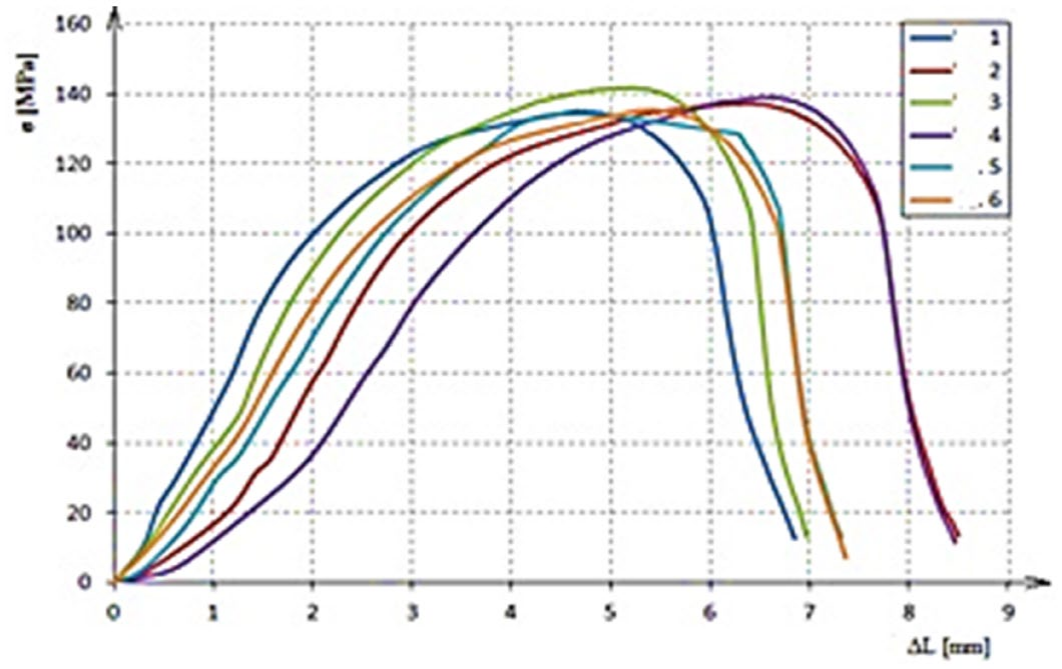
Tensile diagrams.

By a transverse tensile test, some after-welding mechanical properties were ascertained such as ultimate strength (Rm), proof stress (R_p0.2_), and ductility (A). The most observed value is the R_p0.2_ proof stress because, based on this value, an FEM simulation design is subsequently calculated and created. For the door frame, this value is more important than the ultimate strength because the original shape of the door frame must be preserved even after relaxation. A tensile test was performed on six test coupons. The resulting values of the tensile test are listed in Table 6. The stress depending on the absolute elongation of all the six samples as measured in the tensile test is plotted in the tensile diagram (Fig. 5). The limiting value of R_p0.2_ for the experiment and the FEM calculations is taken from sample No.1, that is, R_p0.2_ = 108.31 MPa.

**Table 6:** Mechanical properties from tensile test.

Sample	No. 1	No. 2	No. 3	No. 4	Nos. 5, 6
Rm (MPa)	135.3	137.2	141.7	139.0	139.8
R_p0.2_ (MPa)	108.3	114.6	109.6	117.4	119.8
A (%)	6.25	7.5	6.25	6.25	6.25

## FEM simulation

A total of six different reduced numbers of welds on a specific door frame were simulated. The first simulated was variant no. 1. This variant was currently welded in a cooperating company. This variant serves as a basic one with all others derived from it. All variants considered in the experiment are listed and described in Table 7.

**Table 7:** Description of the FEM simulation variants.

Variant no.	Description
1	The base variant without “roll”
2	The variant of the cooperating company without “roll”
3	The variant without the outside welds without “roll”
4	The base variant with “roll”
5	The variant of the cooperating company with “roll”
6	The variant without the outside welds with “roll”

**Figure 6:**
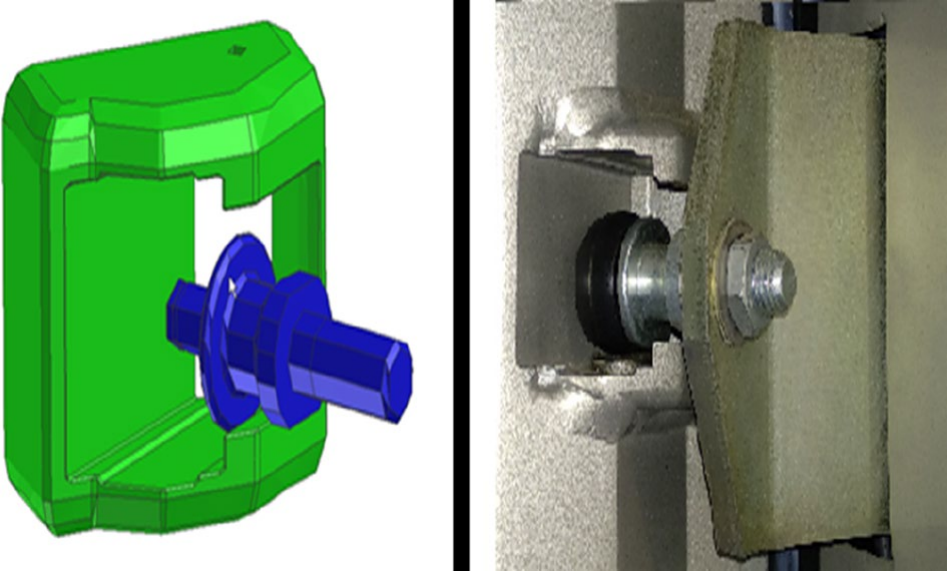
Security backstop (“roll”).

From the simulation of variants 1 to 3, a constructional deviation was detected of the real door frame from the simulated one. The simulation itself did not involve a security backstop (further referred to as roll, (Fig. 6). To increase the precision, the simulation model was modified adding this safety element.

### Improvement of the drawings

**Figure 7:**
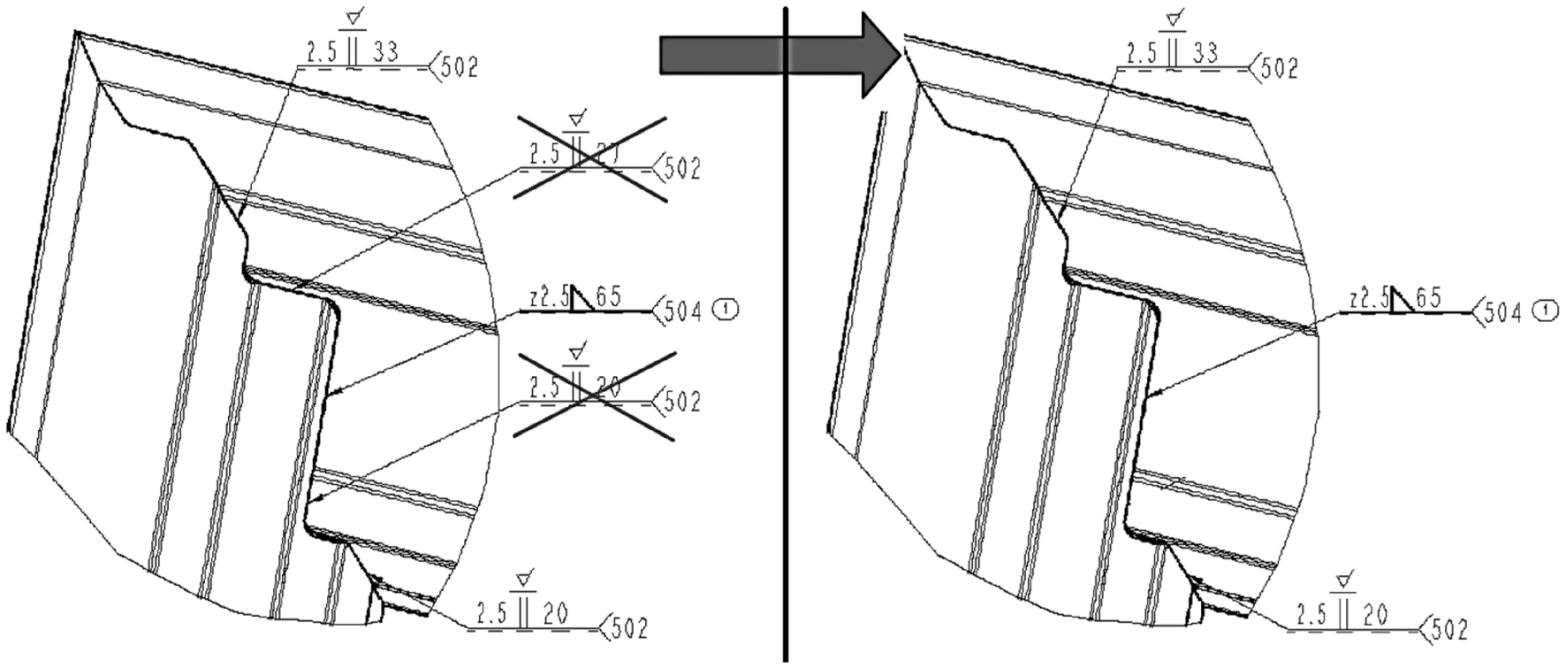
Drawing with detail of the corner of a door frame with reduced welds.

In order to preserve the GTAW welding method, attention was paid to the drawings of the door frame, with respect to individual welds. Reducing the lengths of the welds or leaving out some of them would no doubt shorten the time needed for welding. The thus reduced heat input to the welded frame would lead to minimizing the heat-affected areas, the internal stress, and deformation. This would have a positive effect on productivity bringing down production costs. However, the reduction would need result in less overall toughness of the door frame and a necessity to devise a retrieval method for shielding the gaps brought about by the reduction (Fig. 7). For further frame production, adhesive gap bonding with resulting cementation before powdery painting seems to be a suitable solution.

In principle, reducing the welds must not in any case influence the door frame stability. The requirements that a door construction must strictly meet and observe are defined by the EN 14752c standard. In view of saving finance by reducing the number of loaded door frame prototypes on a real testing table, it was resolved to use software for an FME-based simulation of the load cycle.

### The strength calculation by FEM simulation

The simulation was done by the Virtual Performance Solution (VPS) software. Of the numerous VPM's add-ons, the PAM add-on with Crash extension was used. Virtual Performance Solution is built on the Finite Element Method (FEM). FEM is a computing method used by research and engineering applications solving sophisticated problems which would be difficult to solve by other approaches such as analytical ones. The method's basic idea is to break down complex geometric objects into a finite number of elements (entities) with simple geometry approximating their shapes. Next, the material properties are assigned to each geometric description of a given part. When loaded by external forces applied to geometric shapes, each shape can be described by a system of equations (strength equations or equations describing deformation). Such equations are solved for each entity. The door frame was subjected to two types of loading. The first one was constant load while the second one was a mode analysis. A constant load on the door frame is defined by the EN 14752 standard. The door has to withstand forces caused by passengers being pressed against the door wings without permanent deformation or loss of serviceability. A closed and locked door, including glass covering, has to resist pressing force from the inside on the door wing. The load has to act upon a 100 mm wide band, starting 1300 mm above the doorstep. The value of this force is 1000 N per metre along the width of the inside door surface loaded as shown in Fig. 17. The second load was one given by mode analysis, which is one of the most often used numerical analyses for investigating the dynamic behaviour of a structure. The dynamic behaviour of an excited system can be broken down into independent movements (vibratory modes), inherent to cycle shapes. A mode can be described by modal parameters including the natural frequency, the shape of the natural cycle, the absorbing of a mode and modal weight and stiffness. This method is based on the decomposition of a complex dynamic movement of parts into single modes that, in a real system, represent movements (bend, torsion) loading the structure. Thanks to their exact identification, it is possible to adjust the system design to meet the required parameters. An FEM model was created with the help of shell elements (SHELL) on the central line of Computer-Aided Design (CAD) data. Thus, the input for the Virtual Performance Solution software was a file in the STL format Stereolithography 3D CAD file)^10^.

### Simulation of the calculated variants

**Figure 8:**
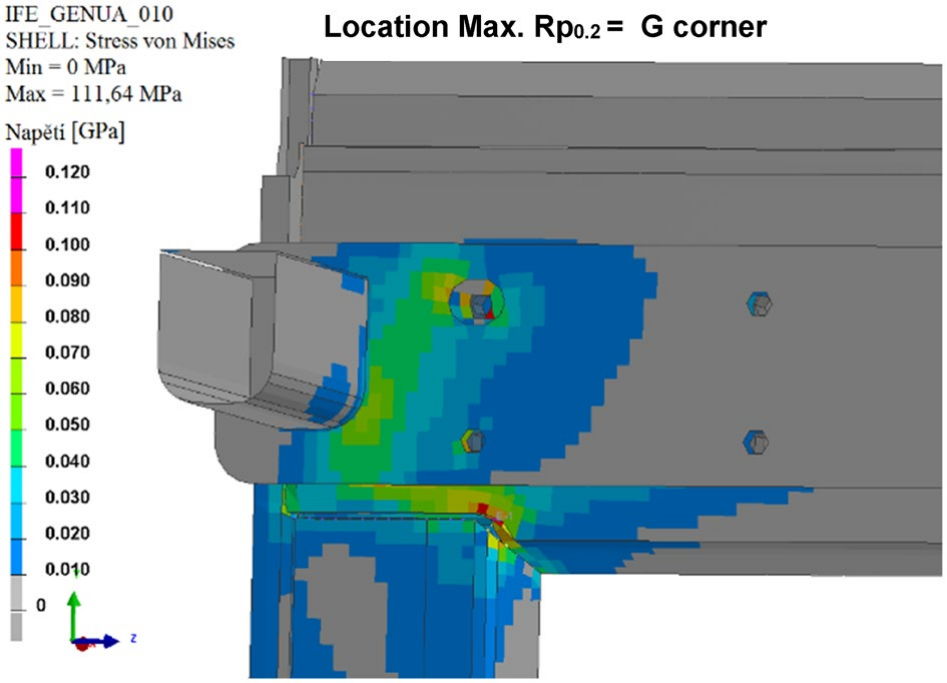
FEM stress map (colour contour) variant no. 1.

**Figure 9:**
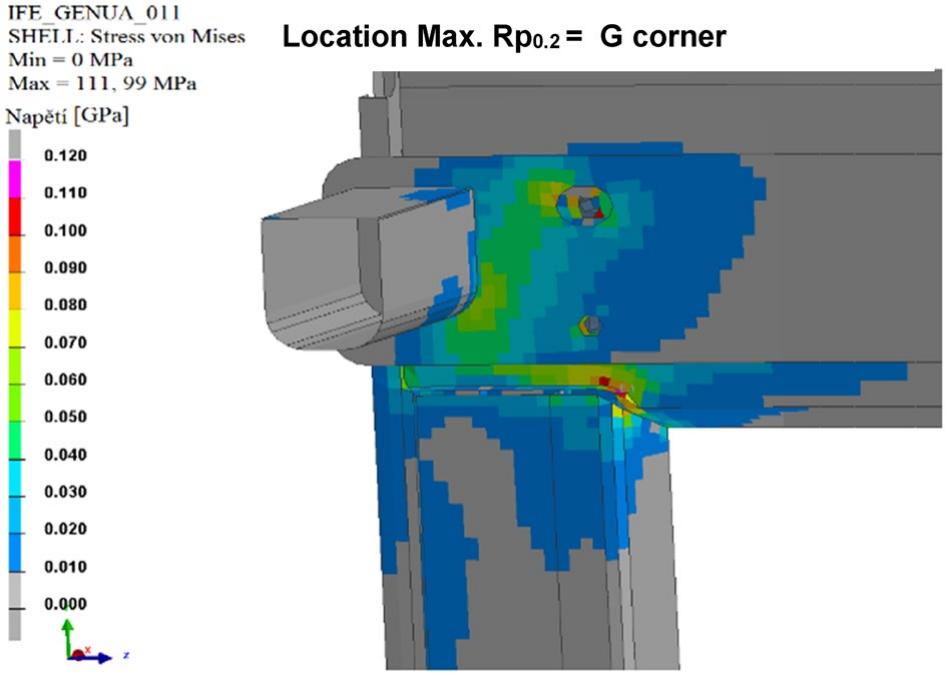
FEM stress map (colour contour) variant no. 2.

**Figure 10:**
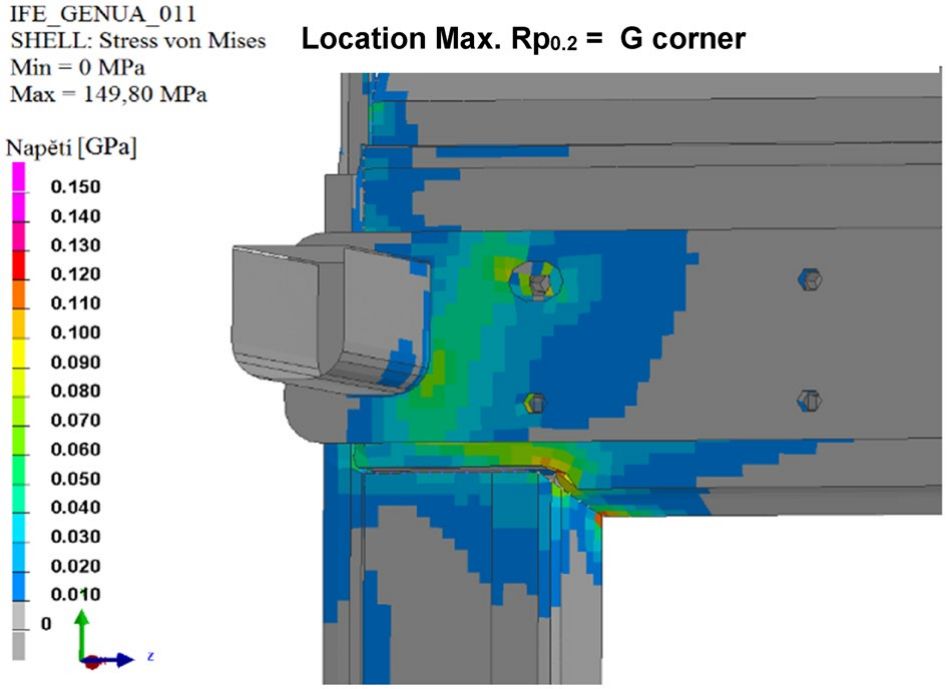
FEM stress map (colour contour) variant no. 3.

**Figure 11:**
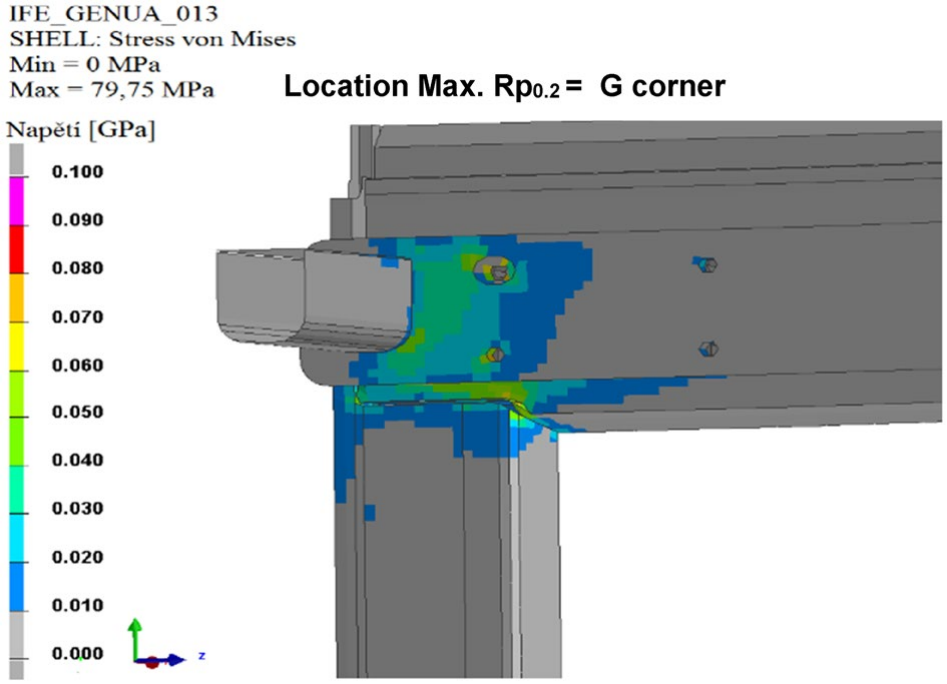
FEM stress map (colour contour) variant no. 4.

**Figure 12:**
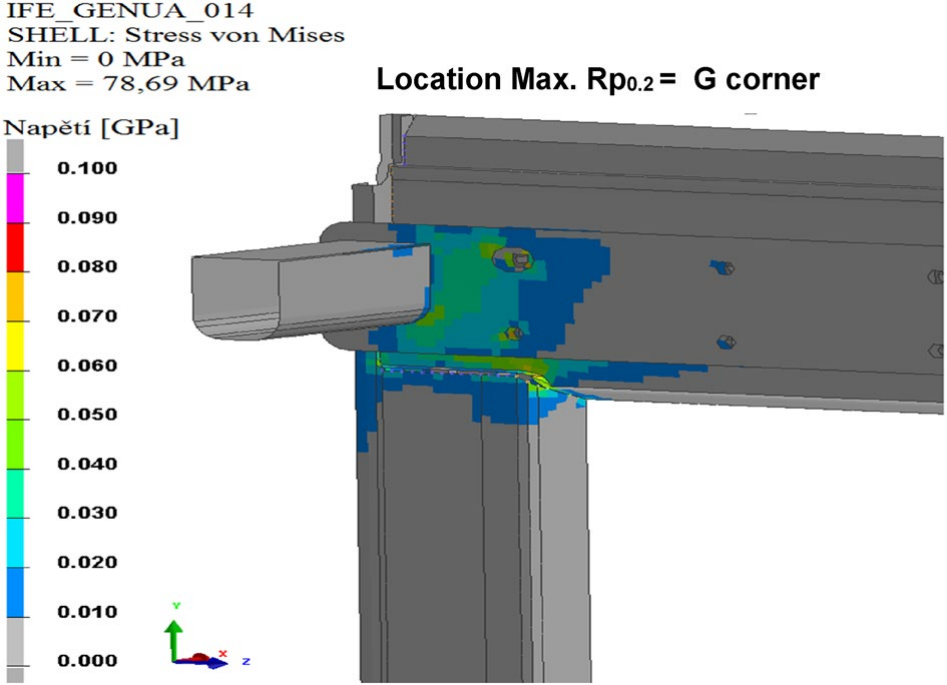
FEM stress map (colour contour) variant no. 5.

**Figure 13:**
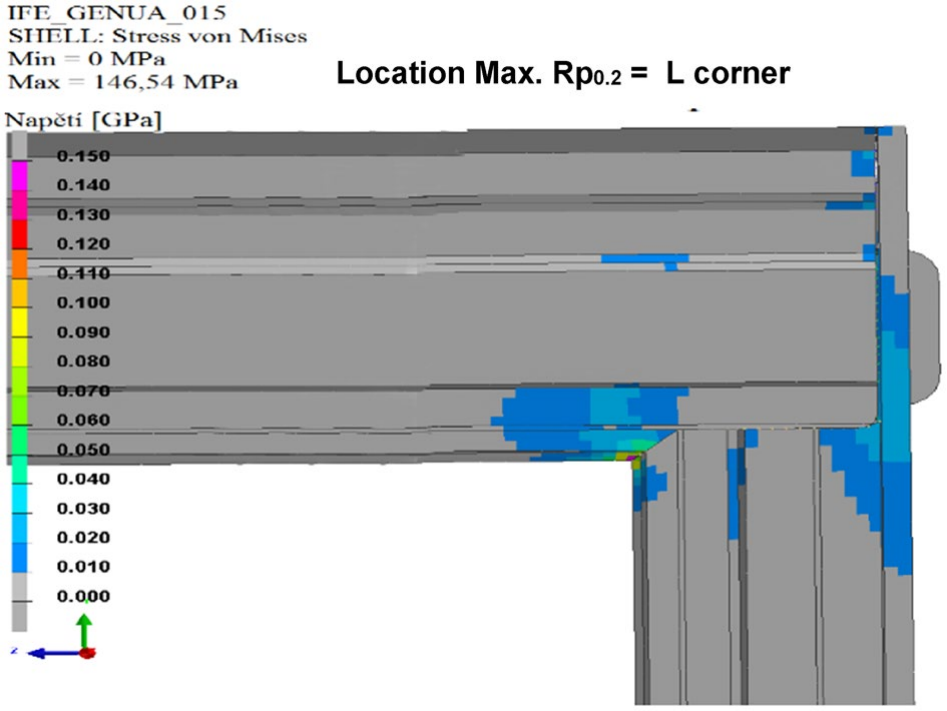
FEM stress map (colour contour) variant no. 6.

The results of the calculated variants are shown below. For each variant, Tables 8, 9, 10, 11, 12, and 13, and Figs. 8, 9, 10, 11, 12, and 13, show maximum stress tension, the stress location, the difference between the maximum stress tension obtained FEM simulation, and its estimate based on a tension test. The R_p0.2_ Limit value is taken from Sample 1, that is, R_p0.2_ = 108.31 MPa. The above maximum stress tensions are always related only to the profiles welded from aluminium alloy EN AW 6060 T66.

**Table 8:** Values of FEM simulation variant no. 1.

R_p0.2_ max. (MPa)	R_p0.2_ limit (MPa)	Difference R_p0.2_ (MPa)	Location max. R_p0.2_
111.64	108.31	**− 3.33**	G corner

Significant value is in bold.

**Table 9:** Values of FEM simulation variant no. 2.

R_p0.2_ max. (MPa)	R_p0.2_ limit (MPa)	Difference R_p0.2_ (MPa)	Location max. R_p0.2_
111.99	108.31	**− 3.68**	G corner

Significant value is in bold.

**Table 10:** Values from FEM simulation variant no. 3.

R_p0.2_ max. (MPa)	R_p0.2_ limit (MPa)	Difference R_p0.2_ (MPa)	Location max. R_p0.2_
149.80	108.31	**− 41.49**	G corner

Significant value is in bold.

**Table 11:** Values from FEM simulation variant no. 4.

R_p0.2_ max. (MPa)	R_p0.2_ limit (MPa)	Difference R_p0.2_ (MPa)	Location max. R_p0.2_
79.75	108.31	**28.56**	G corner

Significant value is in bold.

**Table 12:** Values from FEM simulation variant no. 5.

R_p0.2_ max. (MPa)	R_p0.2_ limit (MPa)	Difference R_p0.2_ (MPa)	Location max. R_p0.2_
78.69	108.31	**29.62**	G corner

Significant value is in bold.

**Table 13:** Values from FEM simulation variant no. 6.

R_p0.2_ max. (MPa)	R_p0.2_ limit (MPa)	Difference R_p0.2_ (MPa)	Location max. R_p0.2_
146.52	108.31	**− 38.23**	L corner

Significant value is in bold.

From the above FEM-simulation output values, it is clear that it is only in variants 4 and 5 that a sufficient strength of the door frame has been achieved. Variant No. 4 is currently welded in a cooperating company. Variant No. 5 is newly designed with the number of welds being reduced by eleven. The maximum stress tension is 78.69 MPa, with a reserve of 29.62 MPa with respect to the limit value. However, such results are based on an FEM simulation so that the real values may be somewhat different. No. 6 cannot be considered successful by any means. The limit value of maximal tension has been exceeded by 38.23 MPa. Although this would be the best variant in terms of saving, the numeric simulation alone proves that this design is by no means good^11^.

## Welding the recommended variant

**Figure 14:**
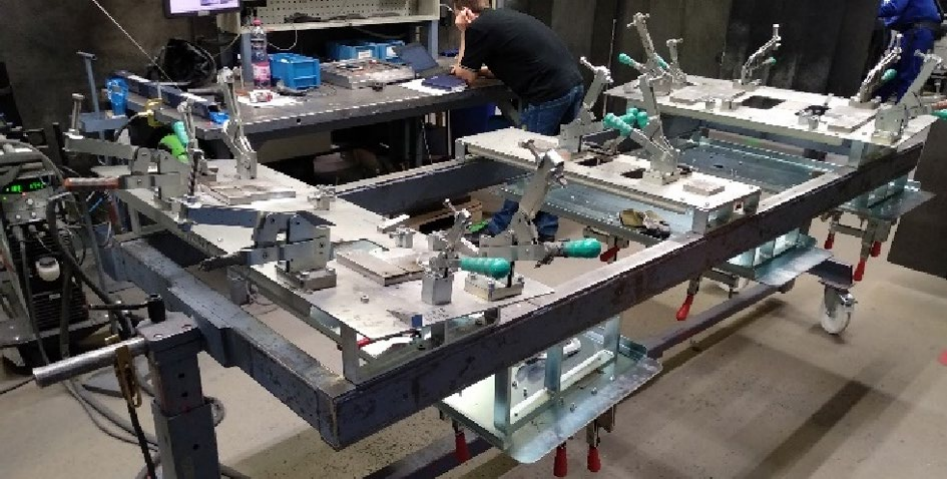
Welding jig for welding a door frame.

The door frame consists of five aluminous profiles, of which four are welded by the GTAW method. Before the actual welding, the profiles are close-set to a welding jig (Fig. 14) to minimize the deformation incurred by the welding, particularly by the heat caused by welding. This welding jig ensures a perfect alignment of the profiles as well as their fixation in a given position. All fixation shoulders are placed on a frame resting on pins so that it can be rotated about the axis. The bogie frame is equipped with transport wheels.

**Figure 15:**
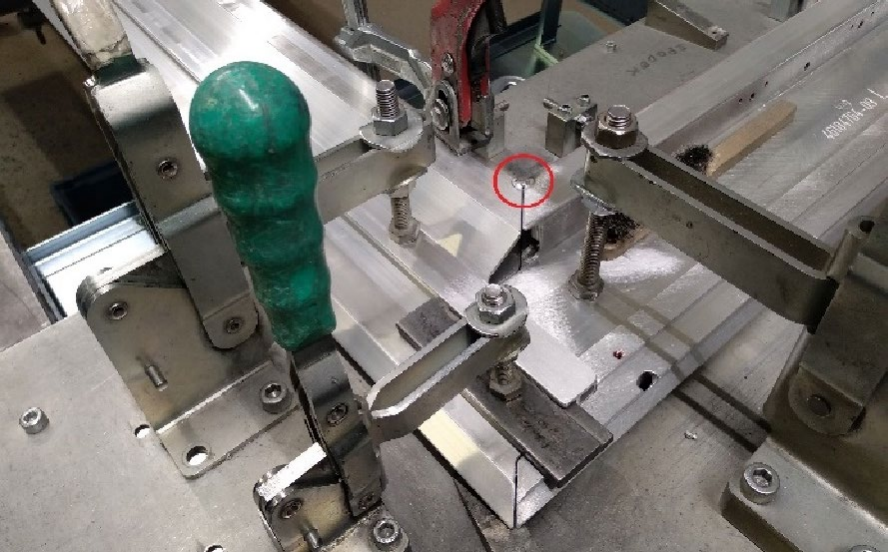
Tack welding of a door frame.

After the profiles are setup, aligned, and clamped into a welding jig, they are tacked together. The tack welding (Fig. 15) is sufficient to ensure the positions of the profiles for welding while the welding heat input is minimal, which prevents deformation of the door frame before the actual welding.

**Figure 16:**
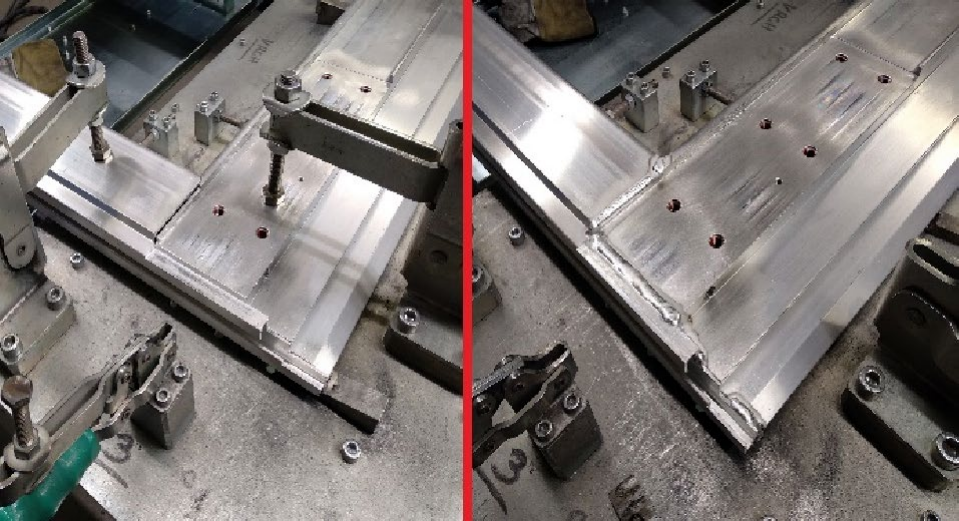
Corner G of the door frame for current variant after welding.

The welding was performed according to the welding procedure specification (WPS) that the welder can see on the monitor in his welding box. First the inner and then the outer sides are welded of the door. Figure 16 is shows corner G of the door frame for the current variant before and after welding. The outside door frame is then welded without a fixture because the door frame already has sufficient toughness.

**Figure 17:**
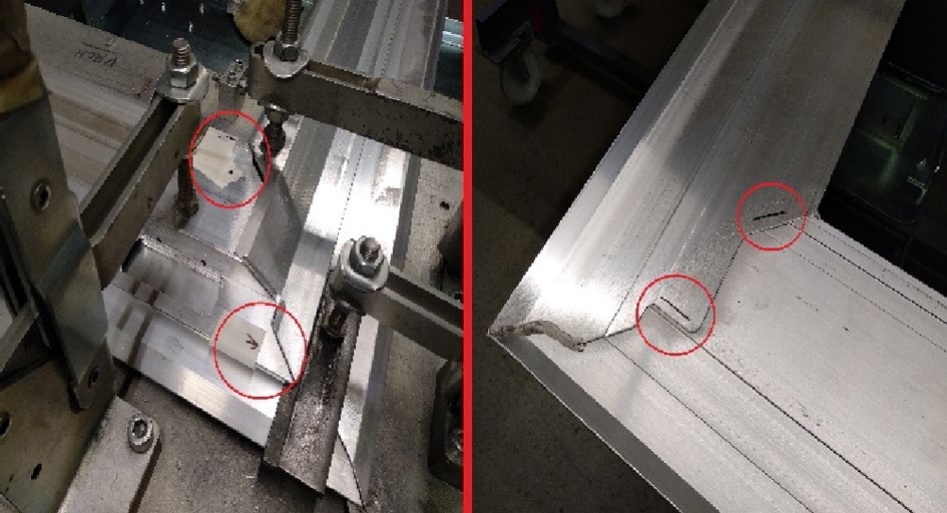
Marking the position of left out welds.

After the current variant, the newly designed one was welded. The steps before and during welding were identical. The only difference was that the welds were not carried out according to the FEM simulation. Figure 17 shows the positions of the left-out welds.

**Figure 18:**
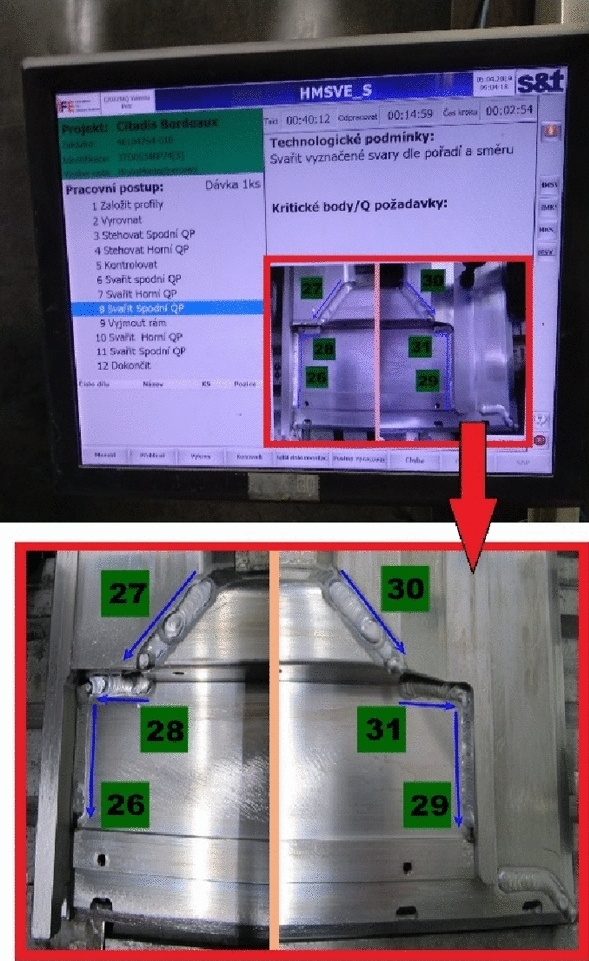
Monitoring of welding process.

The welding procedures of the current and the newly designed door frame were the same. A new design would lead to different heat distribution inside the door frame during welding, which would bring about rising internal residual stresses. Thus, after being released from the welding jig, the welded frame would be deformed necessitating subsequent straightening, extending the total welding time, and increasing the manufacturing time and costs. While working, the welding operator can see the operation on a monitor in his welding box. Figure 18 shows such a monitor showing a particular welding process and a photo with marked welds indicating the direction of welding.

## Experiment evaluation

**Figure 19:**
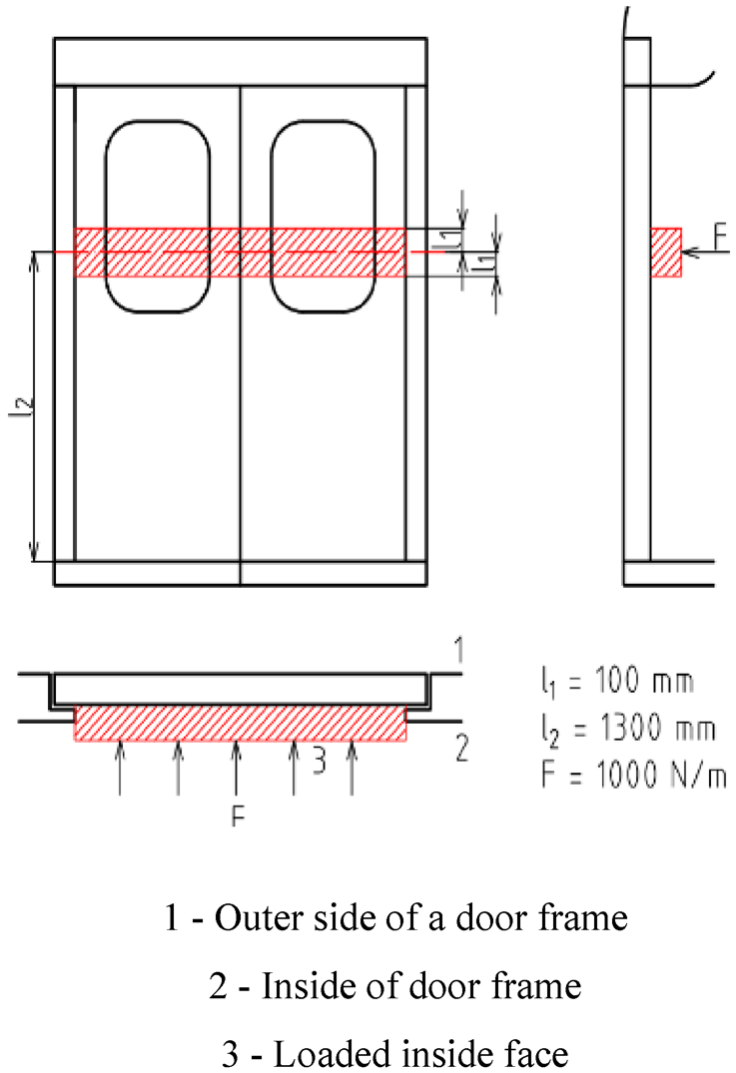
Location of loading during static testing.

**Figure 20:**
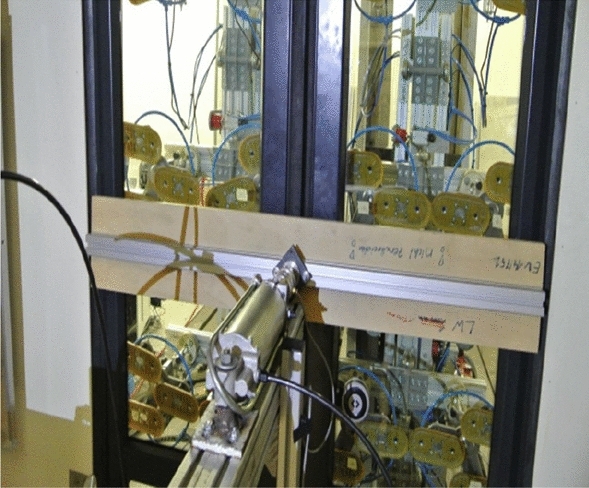
Real loading (static) on the testing table.

**Figure 21:**
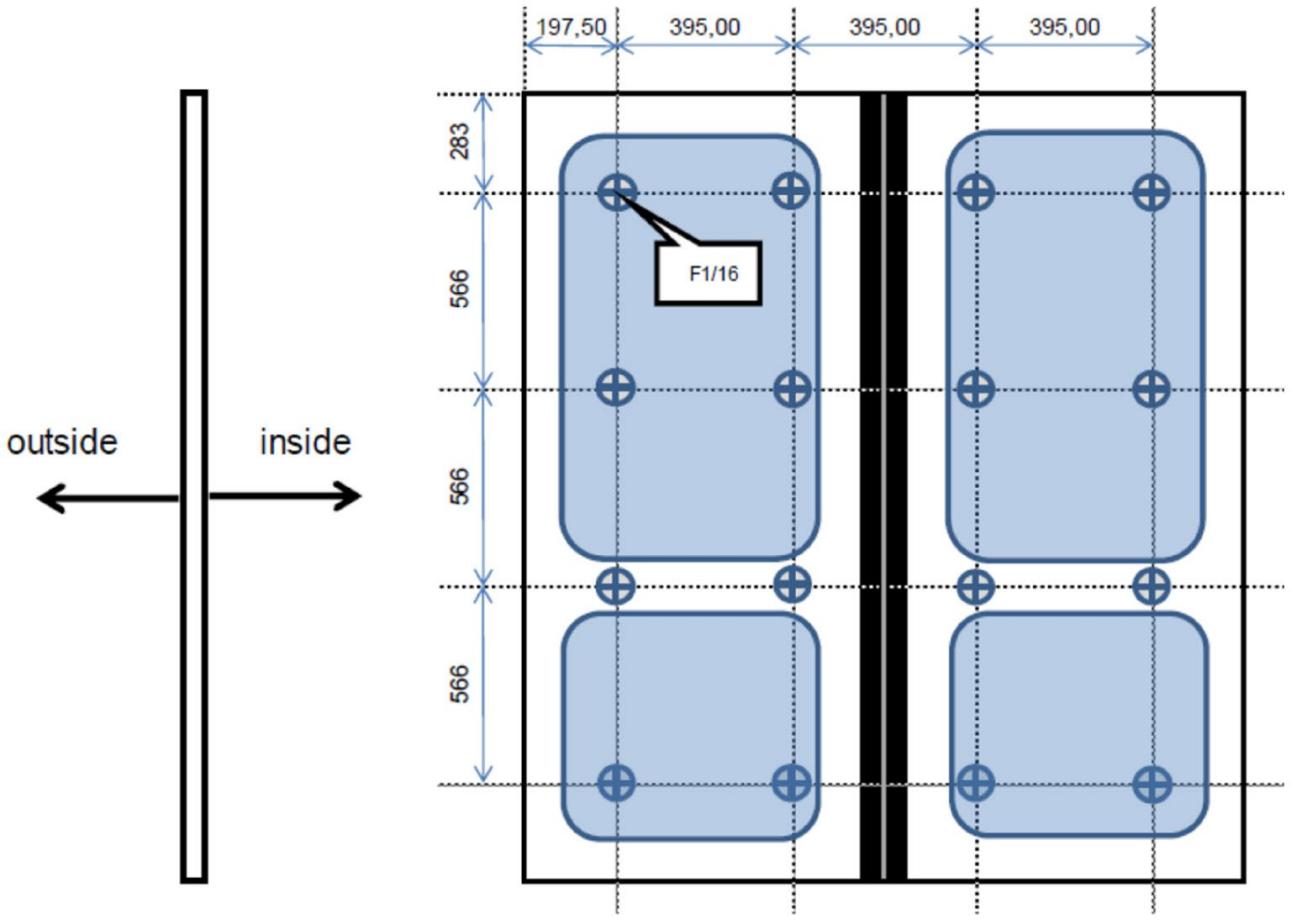
Location of loading during dynamic testing.

**Figure 22:**
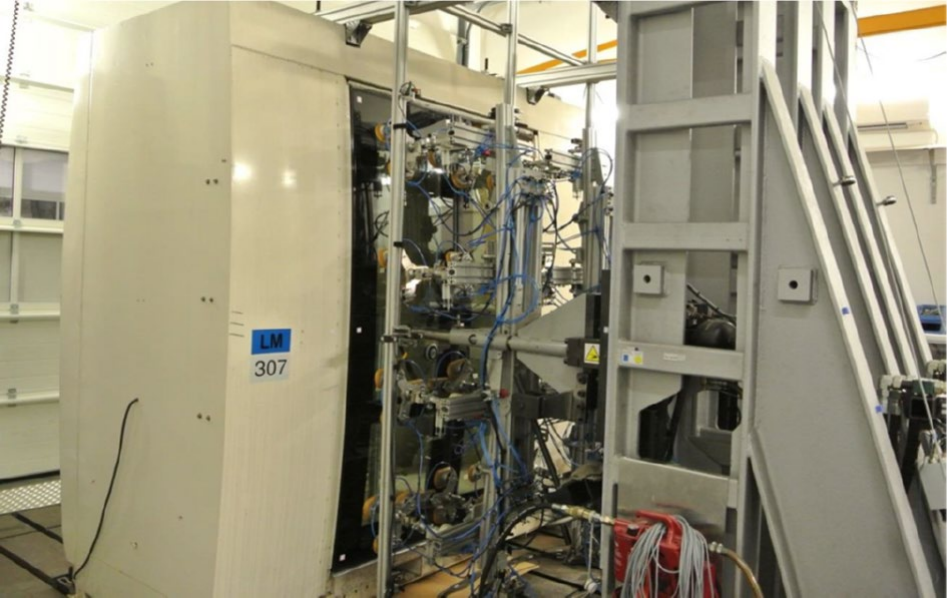
Real loading (dynamic) on the testing table.

In spite of the positive result of FEM simulations, the newly designed door frame with a reduced number of welds cannot be released for production without being tested on a real testing table. The deformation and residual stress tension parameters as calculated by the simulation software are still only indicative. Even if the simulation programs show rapid development, the primary testing method is still a real test. All the loading tests were done by the cooperating company. The given design was tested using loading corresponding to the EN 14752 standard. The following schematic view (Fig. 19) shows how the required force has to be applied to the door. The application of the force takes place with a pneumatic cylinder. The location and amount of loading for static test are shown in Fig. 19. Figure 20 shows the view of a real static testing table. A static test procedures consist from first the door is open and closed manually. Than the force is applied and then removed again so that one-time settlements in the system will not be considered during the measurements. At the measuring points are defined the origin distances are measured. All these testing are repeated three times and at the end a function test of the door system is performed by opening and closing manually several times. The location and amount of loading for dynamic test are shown in Fig. 21. The following schematic view (Fig. 19) shows how the required force has to be applied to the door. The application of the force takes place with the hydro-pulse testing unit with two cylinders. The dimensions contained in the figure are related to the door outer edge (seen from outside). Figure 20 shows the view of a real dynamic testing table. During dynamic load case one is applied force 800 N/m^2^ and total force to door be applied according to standard must stand test without rupture minimally 2862 N. During dynamic load case two is applied force 1450 N/m^2^ and total force to door be applied according to standard must stand test without rupture minimally 5187 N. During dynamic load case three is applied force 1800 N/m^2^ and total force to door be applied according to standard must stand test without rupture minimally 6439 N. A dynamic test procedures consist from first the door is opened and closed manually. After the door was brought to the closed position, the door is measured at the defined points. The hydro-pulse testing unit is docked as shown in Fig. 22. The test with the cycles are defined and the forces are calculated. After the defined cycles were completed, the test will be stopped and the hydro-pulse testing unit is undocked. The test procedure is repeated with the additional load cases^10^. All these static and dynamic testing on welded door frame variant No.5 passed.

## Conclusions

The materials used to manufacture the frame of a rail vehicle have to meet strict requirements over the whole service life starting from production. The door frame of this study consists of profiles made from the EN AW 6060 material, which is thermally processed according to state T66. The required tensile properties were determined using test samples gained from welded plates from the EN AW 6060 T66 aluminous alloy. The results confirmed the deterioration of the mechanical properties caused by the heat from welding and served as the limit values used in an FEM simulation of the loading of a specific door frame. A method was considered of reducing the number of welds to accelerate the manufacturing time of a door frame during the welding cycle. Although reducing the number of welds would no doubt shorten the operation time, it would also influence the strength and toughness of the door frame.

Reducing the number of some welds on the door frame without a previous FEM-based simulation brings about the necessity to repeat the real loading test on the testing table. Such repetition would entail increased production costs. For this reason, the new design was first put to an FEM-based simulation using the Virtual Performance Solution program with PAM—Crash extension. A total of five variants were calculated choosing the one best suited for a new design. The current and newly designed variants were carried out. The newly designed and welded frame was subsequently positively tested on a real testing table, which verified its suitability.

The project about that we report in this scientific technical paper was done during last four years and was finished with results to application in cooperative company. According to basic information from cooperative company produce e.g. 8000–10,000 pcs. aluminium door frame per year. Results and conclusion of this project study of welds reduction save a lot of money a production time and increase productivity of manual welding. Nowadays and in the future during three years with cooperation BUT FME and VUT department of Robotics and IFE-CR, s.r.o., we will cooperate on project robotic welding these aluminium door frame systems. These new project is set up according to knowledge and experiences of project described and practical tested in this study. And the same system from FEM simulation, welding and testing (production) by manual welding will be used also for a robotic welding. We hope that will be used the same system on door frame parts welded on robot. Me and also my development team think that this paper study and of course the technical report is innovative, because don’t exist any similar development project for transportation systems. That was simulated, was produced and tested and used in a service in practice. And at the end help improve system of modelling and testing also for a robotic welding in a practice. System is still after four years using and working and help also in an academic scientific area during my teaching lessons of welding and FEM simulation and help also in the practical sector for a cooperative company.

## Data Availability

All data generated or analyzed during this study are included in this published article. Only the one dataset generated and/or analyzed is not publicly available due [because VB190122 Dynamic/Static Load Test Summary is internal Ife instruction] but are available from the corresponding author on reasonable request.
